# Identifying diseases-related metabolites using random walk

**DOI:** 10.1186/s12859-018-2098-1

**Published:** 2018-04-11

**Authors:** Yang Hu, Tianyi Zhao, Ningyi Zhang, Tianyi Zang, Jun Zhang, Liang Cheng

**Affiliations:** 1 School of Life Science and Technology, Department of Computer Science and Technology, Harbin Institute of Technology, Harbin, 150001 People’s Republic of China; 2 Department of rehabilitation, Heilongjiang Province Land Reclamation Headquarters General Hospital, Harbin, 150001 People’s Republic of China; 3 College of Bioinformatics Science and Technology, Harbin Medical University, Harbin, 150001 China

**Keywords:** Metabolites, Similarity of diseases, Similarity of metabolites, Random walk, InfDisSim, MISIM

## Abstract

**Background:**

Metabolites disrupted by abnormal state of human body are deemed as the effect of diseases. In comparison with the cause of diseases like genes, these markers are easier to be captured for the prevention and diagnosis of metabolic diseases. Currently, a large number of metabolic markers of diseases need to be explored, which drive us to do this work.

**Methods:**

The existing metabolite-disease associations were extracted from Human Metabolome Database (HMDB) using a text mining tool NCBO annotator as priori knowledge. Next we calculated the similarity of a pair-wise metabolites based on the similarity of disease sets of them. Then, all the similarities of metabolite pairs were utilized for constructing a weighted metabolite association network (WMAN). Subsequently, the network was utilized for predicting novel metabolic markers of diseases using random walk.

**Results:**

Totally, 604 metabolites and 228 diseases were extracted from HMDB. From 604 metabolites, 453 metabolites are selected to construct the WMAN, where each metabolite is deemed as a node, and the similarity of two metabolites as the weight of the edge linking them. The performance of the network is validated using the leave one out method. As a result, the high area under the receiver operating characteristic curve (AUC) (0.7048) is achieved. The further case studies for identifying novel metabolites of diabetes mellitus were validated in the recent studies.

**Conclusion:**

In this paper, we presented a novel method for prioritizing metabolite-disease pairs. The superior performance validates its reliability for exploring novel metabolic markers of diseases.

## Introduction

Complex and ordinal chemical reactions in the human body are essential for maintaining human life. The whole process is called metabolites [1, 2]. The maintenance, growth and reproduction of organisms are depended on the metabolites [3]. In terms of gaining energy, metabolites are divided into two sections. One is obtaining energy by the catabolism of large molecules, such as cellular respiration. The other one is getting energy by the synthesis inside the cells, such as proteins and nucleic acids [4]. Once people get sick, the exchange of substances and energy would occur abnormity. Then a series of abnormal metabolites would be generated. Therefore, metabolites can effectively diagnose and treat diseases [5].

Nowadays, recognizing diseases in the molecular level can be achieved by the advanced technology, which is really helpful to the researchers [6–14]. Many researchers aim to find out the role of single gene, single mRNA transcript and protein towards diseases [15]. This leads to a high explanation of diseases. While the complex genes and micro-RNAs often interact with others, it is hard to analysis the underlying mechanism of diseases. However, metabolisms are the final production of the mechanisms, which have already been a significant factor to identify diseases.

Firstly, due to the correlations between different diseases, the similarity of diseases can be calculated depend on genes and their corresponding proteins. For example, the colorectal cancer has a strong relationship with ulcerative colitis, which is reported in the PM Choi’s paper [16]. Achalasia and Parkinson’s disease share similar features to some extent, so SJ Qualman et al. [17] found out the similarity of the two diseases. Furthermore, a various researches have reported the methods to obtain the similarity of diseases. J Li et al. [18] developed a method named DOSim to compute the similarity of diseases, and the method has been packaged into a R-based software package. J Wang et al. [19] proposed a method to calculate the phenotype similarity scores, then the score can be used to obtain the similarity of diseases. Rischer et al. [20] built a gene-to-metabolites network to explain the mechanism of *catharanthus roseus* Cells. Mounet et al. [21] also built a network of genes and metabolites to find out the candidate gene for tomato’s composition and development. To improve the robust of metabolites’ network, Huss [22] divided the network into small subnetworks and removed the most abundant substrates. Based on the 3D-structure similarity of metabolites, Ohtana et al. [23] found out the relationship between biological activities and metabolites. Steve O′ Hagan and Douglas B. Kell [24] analyzed the similarity between drug and metabolites. Kang et al. [25] classified the plants by their metabolites’ similarity.

Since metabolites are the key to explain the diseases’ mechanisms. Analyzing the metabolisms is very attractive to researchers because the number of compounds which are needed to be identified and quantified is relatively low [26]. In 2009, Vladimir V.Tolstikov [27] developed a method that can find out more related metabolites to the data analysis. In 2010, H Zur et al. [28] predicted the enzymes’ metabolic flux by a novel method ‘iMAT’. Paige et al. [29] had collected the metabolisms of depressed patients and did the analysis. M Cuperlović-Culf et al. [30] identified the individual cell lines, groups of cancer and normal cell lines, non-invasive and invasive tumor cell lines by metabolites.

Therefore, we try to find out more related metabolites by analyzing the data of metabolites and diseases. Firstly, we calculated the similarity of different diseases, then the similarity of metabolites could be obtained based on the similarity of diseases, finally a network could be built, where each disease could reach the metabolites on the network. Then we can obtain more disease-related metabolites by the network.

## Methods

### Work frame

**Fig. 1:**
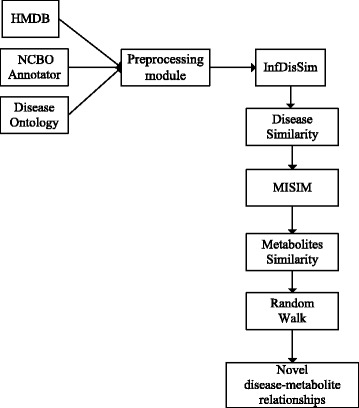
The roadmap of our research work

To clarify the research that we did, a flow chart of our research work is showed in Fig. 1. Firstly, we should get the information of different diseases and metabolites. After getting three data sets, we need to integrate data into a one-to-one corresponding data format between disease and metabolites through a semantic text mining algorithm.Besides, we should also obtain some known metabolites which are related to the diseases. Then the method ‘InfDisSim’ is employed to calculate the similarity of different diseases. After that, the method ‘MISM’ is applied to obtain the similarity of metabolites. Then we could build a network of similarity of metabolites. Finally, we found out some novel disease-metabolite relationships by Random Walk.

To obtain the basic relationship between metabolites and diseases, three datasets are used as following: HMDB, NCBO Annotator and Diseases ontology.

### Data collection and database content

#### Human metabolome database

We downloaded the metabolites data from Human Metabolome Database (HMDB) [31]. The most widely used and complete database involves more than 40,000 kinds of metabolomes. It contains three kinds of data information: Chemical data, Clinical data and Biochemical data. They collected this information from thousands of public sources.

The dataset we got is the diseases’ related metabolites which has many complex files. So we would use the other datasets to future understand these data.

#### Disease ontology

Diseases Ontology [32] started as a part of NUgene project in Northwestern University in 2003. By summarizing other datasets, Diseases Ontology can strongly support the heredity, environmental factor and other inducements of diseases, which help researchers understands diseases better.

Each disease or the concept of the diseases is a node. They all have cross literature comments and a DOID name is given for each disease. The nodes in the lower layer are subclasses or subtypes of the nodes in the upper layer, and the parent-child relationship between the DOID is preserved in the data information. All the diseases are classified into seven groups: diseases caused by environmental origin, diseases caused by infectious agent, diseases of anatomical entity, diseases of behavior, diseases of biological process, hereditary disease, disease syndrome and gene ontology. All the nodes are connected by the Directed Acyclic Graph (DAG).

After obtaining the data of diseases-related metabolites by HMDB, we used the Diseases Ontology to annotate the diseases. Therefore, we can know the name and the related information of the diseases.

#### National center for biomedical ontology

In order to improve the semantic expression ability and open interconnection ability of data, National center for biomedical ontology (NCBO) [33] proposed a data sharing project to solve the lack of integration tools for scientific ontologies. The dataset of each domain are presented in the form of information islands. Most of the information can not be semantically identified by the machine, so that there is an obstacle to the interaction between the information nodes, which goes against to biomedical research and knowledge discovery. NCBO has six core components, including computer science and biomedical informatics research, promoting biology projects and external research collaboration, infrastructure, education, communication and management.

We can further understand and annotate the HMDB data through NCBO. Then a disease-to-metabolic data file can be obtained.

### Method

#### Calculating similarity of pair-wise diseases

There is a certain similarity between diseases, whereas the similarity is often caused by the same molecular origins. Protein-coding genes’ interaction can reflect the mechanism of the diseases to some extent. Therefore, the similarity of diseases can be achieved by the genes behind the diseases.

In this paper, to calculate the similarity of the diseases we used the method named ‘InfDisSim’ [13, 34]. This method measured the similarity of diseases by gene functional network. Gene functional network can provide the information flow which can be used to calculate the disease similarity. To analyze the information flow, ITM Probe [35] is employed which included three models: absorbing, emitting and channel. Each disease is a boundary node in the network, besides, each gene is a transient node.

Each disease has several related metabolites, if the number of the metabolites is N, the weight vector of disease *t*_*1*_ would be:

1 $$ {WV}_{t_1}=\left\{{w}_{1,1},{w}_{1,2},\kern0.5em \dots \kern0.5em ,{w}_{1,i},\kern0.5em \dots \kern0.5em ,{w}_{1,N}\right\} $$

Here ${\mathrm{WV}}_{t_1}$is the weight vector of *t*_*1*_, *w*_1, *i*_the weight score of *t*_*1*_ on the *i*th dimension. The cosine of their vectors is used to represent the disease similarity, the equation is as following:

2 $$ Inf\left({t}_1,{t}_2\right)=\frac{\sum \limits_{i=1}^N{w}_{1,i}\cdot {w}_{2,i}}{\sqrt{\sum \limits_{i=1}^N{w^2}_{1,i}\sqrt{\sum \limits_{j=1}^N{w^2}_{2,j}}}} $$

The similarity of disease is defined as following:

3 $$ InfDisSim\left({t}_1,{t}_2\right)= Inf\left({t}_1,{t}_2\right)\frac{\left|{G}_1\right|\left|{G}_2\right|}{{\left|{G}_{MICA}\right|}^2} $$

Where*G*_1_,*G*_2_ indicates metabolites set of *t*_*1*_ and *t*_*2*_, respectively. *G*_*MICA*_is the metabolites set of *t*_*3*_. And ∣. ∣ represents the number of terms in the specified set.

Then we could obtain the similarity of the diseases.

#### Calculating similarity of pair-wise metabolites

A method named ‘MISIM’ was proposed by Dong Wang et al. [36] which is used to estimate the similarity of micro-RNAs. In the research, they pointed out that the genes which have similar functions are often associated with similar diseases, so the similarity of diseases could be computed by DAG. This idea is quite similar with the work we did in the ‘InfDisSim’, in addition, this is also the premise of calculating similarity of metabolites. Due to the thought and the miRNA-disease association data, they presented ‘MISM’ to infer the functional similarity of miRNAs by the diseases relationship.

Compared with our research, we tried to compute the similarity of the metabolites. Since the background and theoretical basis are the same, we applied the ‘MISM’ to calculate the similarity of metabolites by the similarity of diseases.

Firstly, the semantic similarity which is the relationship between diseases should be defined. Then the similarity of disease to one group of diseases can be calculated as follows:

4 $$ S\left(d,D\right)=\underset{1\le i\le k}{\max}\left(S\left(d,{d}_i\right)\right) $$

Here *d* represent one disease and *D* means one disease group. *S*(*d*, *D*) is the maximum similarity between one disease and one disease set.

After getting the similarity of diseases, we could calculate similarity of metabolites. *D*_1_ involves *m* diseases and *D*_2_ involves *n* diseases. If *D*_1_ is one metabolite which is related to the group of disease and *D*_2_ is another metabolite which is related to another group of diseases, the similarity of the two metabolites could be computed by:

5 $$ Similarity\left({M}_1,{M}_2\right)=\frac{\sum \limits_{1\le i\le m}S\left({d}_{1i},{D}_2\right)+\sum \limits_{1\le i\le n}S\left({d}_{2i},{D}_1\right)}{m+n} $$

Then similarity between *M*_1_ *and M*_2_could be obtained.

#### Predicting novel disease-metabolite relationships using random walk

Random Walk is an important part of stochastic process. For example, if an ant starts from *X*_*t*_, it takes a step forward by the probability of 0.5 (*X*_*t* + 1_ = *X*_*t*_ + 1) or takes a step back by the probability of 0.5 (*X*_*t* + 1_ = *X*_*t*_ − 1). Then the points which the ant arrives at each moment can constitute a one-dimensional random walk process.

Random walk can be regarded as a special case of Markov chain. In the case of current knowledge and information, the past (the historical state) is irrelevant to the prediction of the future (the future state). At each step of the Markov chain, the system can change from one state to another or maintain the current state according to the probability distribution. The change of the state is called transfer, and the probability associated with different states is called the transition probability. If G is the adjacency matrix of graph A, we can normalize A as following:

6 $$ P={D}^{-1}A $$

*D* is the degree matrix of A which is a diagonal matrix. The diagonal element is *D*(*i*, *i*) =  ∑ *A*(*i*, *j*). Here *P* is the random walk matrix, and the sum of the jump probabilities of each node and all other nodes is 1.

A random walk matrix corresponds to a Markov chain, that is, any two states can reach each other. Starting from an arbitrary state, the probability at the next state is as following:

7 $$ {A}_{t+1}={A}_tP $$

The process keeps moving, and after a certain period of time, equilibrium state is reached. The equilibrium state means that the probability distribution of the state is no longer changing. The method to calculate equilibrium state is as following:

8 $$ \pi =D\left(i,j\right)/\sum \limits_i\sum \limits_jA\left(i,j\right) $$

When *πP* = *π*, the equilibrium state is reached.

The basic matrix of Markov chains is defined as:

9 $$ Z={\left(I-P-W\right)}^{-1} $$

Where *I* is a unit matrix, *P* is the corresponding random walk matrix, and *W* is a matrix which the equilibrium state’s rows are stacked. For a regular Markov chain, *W* can be considered as the case where n in *P*^*n*^ tends to infinity.

The algorithm flow is as following:

**Table:** 

Step 1:	
Given initial iteration point x, step length is *λ*, control accuracy is ℓ	
Step 2: Iteration times is N, k is the current iteration time	
Step 3: When k < N, randomly generate a N-dimension vector *u* = (*u*_1_, *u*_2_ … *u*_*n*_).then finish the first walk*x*_1_ = *x* + *λu*^'^	
Step 4: If *f*(*x*_1_) < *f*(*x*), k = 1 and return to the step 2, else k = k + 1 and return to the step 3.	
Step 5: If the optimal solution is not found in N times, the optimal solution is centered on the current optimal solution.	

RWR is a global network ranking algorithm. In terms of the probabilities of the edges between the two nodes, one or several seed nodes can randomly transit to their neighbor nodes. The probability of returning to the start seed node is supposed as γ, and then RWR algorithm can be defined as follows:

10 $$ {P}_{t+1}=\left(1-\gamma \right){AP}_t+\gamma {P}_0 $$

Here, *A* is the column-normalized adjacency matrix, *P*_0_is the initial probability vector and *P*_*t*_ is the probability vector which element at node i at step t. According to the previous study, γ would be 0.85 [37].

**Fig. 2:**
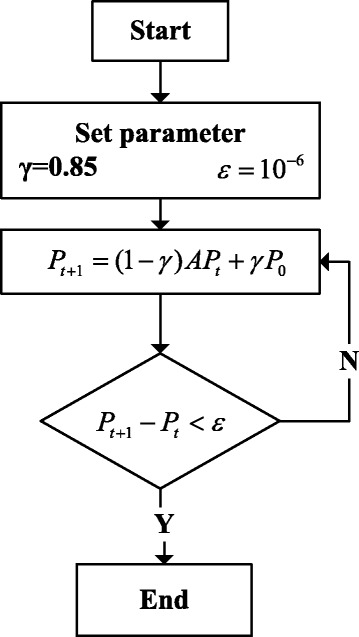
Random Walk on Identifying Diseases-related Metabolites

The Fig. 2 shows the calculation process of Random Walk of identifying diseases-related metabolites. Firstly, we should set parameters, then start the circle until the difference between *P*_*t* + 1_ and *P*_*t*_ is lower than the threshold. Finally, we could get all the possible diseases-related metabolites.

## Results

### Data analysis

First, we use NCBO Annotator and Disease Ontology to process the data we get in HMDB. Then the data would be integrated by metabolites and disease one by one. Finally, we made a statistic of the corresponding diseases and metabolites.

**Fig. 3:**
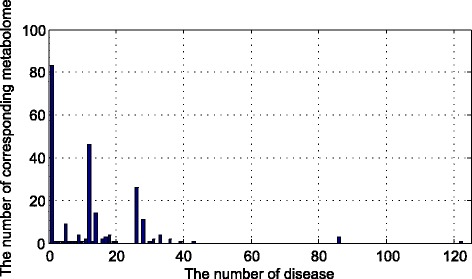
The density of the numbers of correlated metabolomes for one disease

As we can see in the Fig. 3, most of the diseases are related to only a few metabolites. There are 122 diseases that only related to one metabolite. However, for some complex diseases, the number of the corresponding metabolites is quite high, for example, there is a disease that related to more than 80 metabolites. Here we made a hypothesis that most of the diseases should be related to more metabolites.

**Fig. 4:**
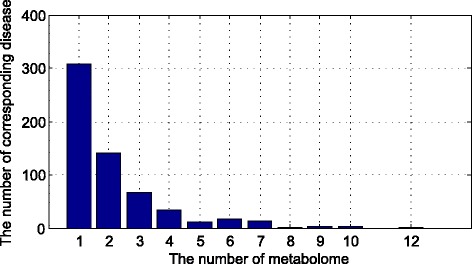
The density of the numbers of correlated disease for one metabolomes

In the Fig. 4, there is a common metabolite which is related to more than 300 diseases. And about 150 diseases are related to two same metabolites. Several various diseases are related to the same 12 metabolites.

After analyzing the two figures, we could speculate that there are more metabolites related to the diseases. To understand the mechanism of diseases, we need to know all the related metabolites.

### The metabolites related to diseases

Further, we calculate the similarity of diseases by InfDisSim. We totally get 3524 diseases and we calculated the similarity between each two diseases.

**Fig. 5:**
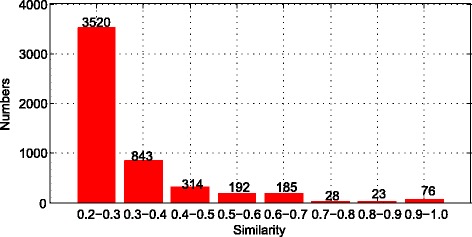
Statistics of the number of different similarities

Since there are 3524 diseases, so we totally get 6,211,050 similarities. In these similarities, most of them are lower than 0.1. To be more precisely, the number is 5923125. In addition to that, 99.92% of the similarities are lower than 0.2. Then we excluded these similarities, and use the rest similarities to draw the Fig. 5. As we can see in the Fig. 5, most of the diseases’ similarities are lower than 0.3. The number of similarities which are higher than 0.5 is quite small. Using these similarities, we could calculate the similarity of metabolites.

**Fig. 6:**
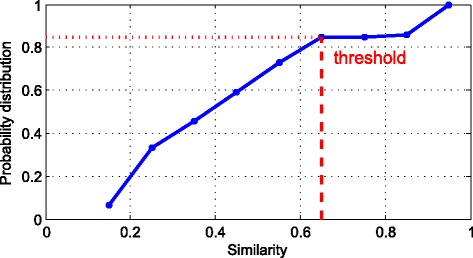
The probability distribution of metabolomes’ similarity

Then we calculate the similarity of metabolites by MISM. In terms of the similarity of metabolites, we could draw the figure as following:

We totally get 604 metabolites, so we get the 182,710 similarities from these metabolites. Among these similarities, 90.8% of them are lower than 0.1. Therefore, we use the rest similarities which are higher than 0.1 to draw the Fig. 6. As we can see in the Fig. 6, very few similarities are higher than 0.7. Every point of the figure means the probability between two points on x axis. Take the first point as an example, about 10% of the rest similarities are higher than 0.1 and lower than 0.2. Due to the huge amount of similarities, we need to filter the similarities which are lower than 0.7. So 0.7 is the threshold to select similarities. Therefore, we excluded more than 90% of the rest similarities to continue the rest research. The number of similarity we collected is 2589.

**Fig. 7:**
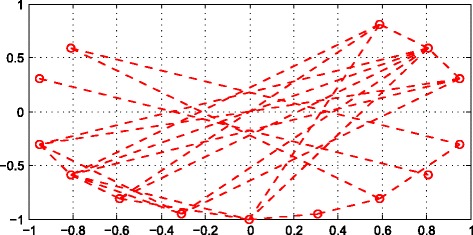
The network of 20 metabolites

There are totally 453 metabolites in these 2589 similarities. Therefore, the network should have 453 nodes, while the figure would be too huge to show in the paper. To show the network we build up, we selected 20 of these metabolomes to draw the Fig. 7.

We distributed 20 nodes in a circle whose radius is 1, and connected them by lines in terms of their similarity. Each note represents a metabolite in the network. If there is relationship between the two nodes, they would be connected by the lines. on the contrary, if the two nodes do not have similarity, they would be divided.Through the lines of the network, diseases can be linked to more metabolites through several known metabolites. In terms of the lines, we could get every metabolite’s probability. We can sort this probability and obtain the candidates of diseases-related metabolites.

After building up the network of 453 metabolites, we use RW algorithm to get the metabolites related to the 228 diseases. For every disease, they may only relate to several metabolites in the known dataset. By the network, we could identify more related metabolites towards every disease.

**Fig. 8:**
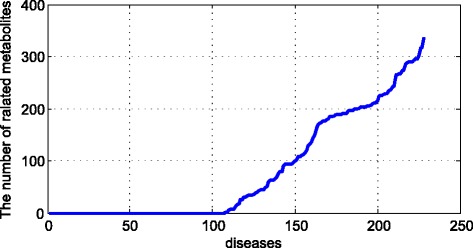
The diseases related to metabolites

As we can see in the Fig. 8, we sorted the diseases by the number of related metabolites. Since we excluded most of the original metabolites, more than 100 diseases could not be found by the related metabolites. Then we can find out more related metabolites to the rest diseases.

**Fig. 9:**
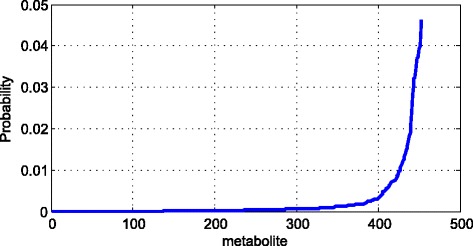
The related metabolites to Alzheimer’s disease

For example, the Alzheimer’s disease is related to 86 metabolites in our original dataset. But we do not know which metabolite has the strongest relationship with it and we also do not know the important degree of different metabolites to this disease. After processing the RW, we could get the rank of metabolites as the following figure:

As we can see in the Fig. 9, there are more than 300 metabolites related to the Alzheimer’s disease. Since Alzheimer’s disease is so complex that we could not precisely know the rank of related metabolites. By this way, we could estimate the rank and analysis the important metabolites.

### Performance evaluation using leave-one-out validation

To validate the performance of our method for prioritizing the metabolite-disease pair, the leave-one-out validation method was utilized here based on existing metabolite-disease associations. Step 1, one metabolite-disease pair was removed from prior knowledge. Step 2, the metabolite network was constructed based on the remained metabolite-disease associations. Step 3, the removal metabolite-disease pair was defined as positive group (PG), and other pairs of metabolites and this disease not in the prior knowledge were defined as negative group (NG). Step 4, we utilized the RWR method to score all the metabolites and disease in the PG and NG based on the network. Step 5, the above steps was iterated for all the metabolite-disease pairs in the prior knowledge. The area under the receiver operating characteristic cure (AUC) was then calculated to validate the performance of our method based on all the NGs and PGs. The high AUC (0.7048) validate the superior performance of our method for predicting novel metabolite-disease associations.

### Case study

Since we mapped the metabolites to the diseases, we found more metabolites which are related to the diseases. To prove the relationship that we found is correct, we conducted a case study.

A good case in point is diabetes mellitus, it is originally related to 28 metabolites, and we found it related to 242 metabolites. Although some of these metabolites’ relationships with diabetes mellitus are weak, there must be some connection between diabetes mellitus and metabolites for sure.

To verify the novel relationship, we selected one of the novel related metabolites to explore whether it is related to the diabetes mellitus. We selected HMDB004793-Methylhistidine which is not reported in the dataset we used in section 2(A). Kuan-Hsing Chen et al. [38] have found this metabolite is related to diabetes mellitus.

DPK Ng et al. [39] have reported that Hydroxyphenylacetic acid is related to the diabetes mellitus. Whereas the original database did not include these metabolites as a related metabolites of diabetes mellitus, we found the relationship between Hydroxyphenylacetic acid and diabetes mellitus by RW.

These two evidences proved that our method is suitable and effective to identify relationship between diseases and metabolites.

## Discussion

We got the data from three public datasets: HMDB, Diseases Ontology and NCBO. Then we got the data which metabolites and disease are one–to-one correspondence. Firstly, we observed the situation that metabolites map to the diseases. Then we speculate that there should be more metabolites that are related to the diseases.

Firstly, we used the ‘InfDisSim’ to calculate the similarity of the diseases. By the genes related to the diseases, we could get the similarity of diseases. Then the similarity of metabolites could be obtained by the similarity of the diseases. The ‘MISM’ gives us a chance to build up a network of metabolites’ similarities. Finally, we used the Random Walk to find more metabolites which are related to the diseases.

By the network of metabolites’ similarity, more metabolites could be connected to the diseases by the lines. The correlation coefficient between the diseases and metabolites could also be obtained. Then we could sort these scores and understand which metabolites are most likely to be associated with disease and which ones are less related to the diseases. The rank could be the important information for researchers to find out the candidate metabolites. The researchers should not be limited by the metabolites reported, the complex metabolites network might give them more chances to understand the mechanism behind diseases.

The presented approach in this paper is also used to predict central nervous system disease-related SNPs and risk pathways by constructing virtual SNP-SNP network and pathway-pathway network [12, 40–43].

## Conclusions

The complex diseases are caused by complex gene interactions. It is hard to explain the mechanism behind diseases by these complex gene networks. However, the corresponding micro-RNAs may not fully explain the way diseases work. Metabolites, as a production of the complex mechanism have become the vital factor to understand the diseases.

The result shows the power of our method and it would be helpful to the further research. We found the unreported metabolites which are related to diabetes mellitus are reported in other researchers’ works. Through our network, these unknown metabolites could be mapped to the diseases.
